# Minnesota

**Published:** 1896-05

**Authors:** 


*Minnesota.* The Bowberg milk-case brought out a strong array of witnesses to the value of tuberculin as a diagnostic agent and the absence of danger in its use. Veterinarians Cotton, Reynolds, and Hess, and Dr. Hewitt, of the State board of health, all gave testimony on these points. The inspection of milk and dairies for Minneapolis has done an unlimited amount of good for that city in producing a purer and better milk-supply for her people.

Only one weak feature of the inspection laws exists, which it is hoped will be remedied by the next legislature, so that immediate destruction may be enforced of all animals suffering with tuberculosis.

